# A retrospective comparison of induction chemoimmunotherapy versus chemotherapy followed by concurrent chemoradiotherapy and consolidation immunotherapy in stage III non-small cell lung cancer

**DOI:** 10.3389/fonc.2024.1432954

**Published:** 2024-10-08

**Authors:** Jing Zhao, Da Miao, Jiaqi Zhou, Siyu Guo, Yang Tang, Fen Lan, Lixia Xia, Ting Zhang, Jian Huang

**Affiliations:** ^1^ Department of Medical Oncology, Second Affiliated Hospital, Zhejiang University School of Medicine, Hangzhou, Zhejiang, China; ^2^ Key Laboratory of Tumor Microenvironment and Immune Therapy of Zhejiang Province, Cancer Institute, Second Affiliated Hospital, Hangzhou, Zhejiang, China; ^3^ Key Laboratory of Cancer Prevention and Intervention, Cancer Institute, Ministry of Education, The Second Affiliated Hospital, Zhejiang University School of Medicine, Hangzhou, Zhejiang, China; ^4^ Key Laboratory of Respiratory Disease of Zhejiang Province, Department of Respiratory and Critical Care Medicine, Second Affiliated Hospital, Zhejiang University School of Medicine, Hangzhou, Zhejiang, China; ^5^ Department of Oncology, Shaoxing Second Hospital, Shaoxing, Zhejiang, China; ^6^ Key Discipline of Jiaxing Respiratory Medicine Construction Project, Jiaxing, Key Laboratory of Precision Treatment for Lung Cancer, Department of Respiratory, The Affiliated Hospital of Jiaxing University, Jiaxing, Zhejiang, China; ^7^ Department of Radiation Oncology, Second Affiliated Hospital, Zhejiang University School of Medicine, Hangzhou, Zhejiang, China; ^8^ Cancer Center, Zhejiang University, Hangzhou, Zhejiang, China; ^9^ Department of Breast Surgery, Second Affiliated Hospital, Zhejiang University School of Medicine, Hangzhou, Zhejiang, China

**Keywords:** non-small cell lung cancer, locally advanced, induction therapy, immune therapy, radiotherapy

## Abstract

**Background:**

Patients with locally advanced non-small cell lung cancer (LA-NSCLC) usually bear high tumor burden and are not tolerated well to concurrent chemoradiation therapy (CRT) followed by consolidation immunotherapy. We investigated the feasibility of chemoimmunotherapy as induction therapy before CRT for LA-NSCLC.

**Methods:**

We retrospectively analyzed data from 91 patients with unresectable stage III NSCLC treated with either induction chemoimmunotherapy or chemotherapy before CRT. Tumor responses, survival statistics, and toxic effects were compared. The dosimetric parameters of the RT protocol were evaluated. The primary endpoint was progression-free survival (PFS). The overall response (ORR), the depth of response (DpR) were accessed at the end of CRT (ORR_induc+CRT_, DpR_induc+CRT_) and induction therapy (ORR_induc_, DpR_induc_).

**Results:**

The median PFS (mPFS) were significantly longer in the chemoimmunotherapy induction group (13.5 months vs. 11.2 months; HR, 0.56; 95% CI, 0.32–0.97; p=0.036). The ORR_induc+CRT_, median DpR_induc+CRT_ (mDpR_induc+CRT_) and mDpR_induc_ were significantly higher in the chemoimmunotherapy induction group (ORR_induc+CRT_, 84.0% vs. 65.9%, p=0.044; mDpR_induc+CRT_, 49.5% vs. 39.0%, p = 0.012; mDpR_induc_, 38.5% vs. 28.0%, p=0.044). Incidence of treatment-related adverse events (AE) was similar between groups, with myelosuppression being the most common grade ≥ 3 AE. Regarding radiotherapy, adopting a mapping strategy with a 5–8 mm margin for clinical tumor volume resulted in decreased radiation doses to critical organs in the chemoimmunotherapy induction group.

**Conclusions:**

Chemoimmunotherapy induction therapy before CRT improves efficacy with comparable incidence of AEs compared to chemotherapy induction in LA-NSCLC patients. Further studies are warranted to validate these findings.

## 1 Introduction

Lung cancer is the second most common cancer type worldwide and the leading cause of death. Non-small cell lung cancer (NSCLC) accounts for ~85% of the total lung cancer population (1). Despite the broad adoption of early screening, 30% of NSCLC patients will have locally advanced (LA) stage III disease at the time of the initial diagnosis, a majority of which present with large tumor loads and unresectable disease (2). Despite the absence of distant metastasis, the prognosis of LA-NSCLC is generally poor. The PACIFIC trial redefined the standard of care for patients with unresectable LA-NSCLC that it currently consists of immune checkpoint inhibitor (ICI) maintenance with durvalumab after concurrent chemoradiation therapy (CRT). The median PFS was 16.8 months in the durvalumab group vs. 5.6 months in the control group. The median overall survival (OS) of patients treated with this protocol was 47.5 months (3, 4).

Nevertheless, challenges remain regarding implementation of the PACIFIC protocol in LA-NSCLC patients. In the real-world setting, patients with unresectable LA-NSCLC are heterogeneous with varied tumor burdens. Given their frequently high tumor burden, the enlarged target volume of RT may result in increased toxicity (5), making it impossible to complete subsequent consolidation therapy. In the PACIFIC trial, 15.4% of patients discontinued treatment due to adverse events (AEs) (4). Meanwhile, the incidence of grade 3 pneumonia was as high as 14.3% (6) in the real word according to the PACIFIC model. The low completion rate of standard consolidation ICI may further affect the survival rate. Therefore, in order to decrease the target volume of RT, induction systemic therapy before definitive CRT to achieve maximal tumor downsizing is recommended (7, 8).

The results from the NADIM II and CHECKMATE-816 studies demonstrated impressive tumor reduction and survival benefit in LA-NSCLC patients who received chemoimmunotherapy, which was shown to be superior to chemotherapy (9, 10). Chemoimmunotherapy might therefore comprise a modified form of induction therapy to be administered prior to CRT. The PACIFIC-2 trial evaluated the use of durvalumab initiated concurrently with concurrent chemoradiotherapy (cCRT), followed by consolidation durvalumab, in comparison to cCRT alone for patients with unresectable stage III NSCLC (11). The results indicated that the concurrent approach did not improve the survival but increased the incidence of AEs. This highlights the urgent need to effectively integrate immunotherapy with CRT for patients with unresectable disease to enhance survival outcomes without increasing AEs. Herein, we explored a modified treatment protocol for LA-NSCLC aimed at reducing tumor burden before radiotherapy. This protocol involves induction chemoimmunotherapy prior to CRT, cessation of immune checkpoint inhibitors (ICI) during CRT, followed by ICI consolidation. We conducted a retrospective cohort study to compare the efficacy and safety of the chemoimmunotherapy induction versus chemotherapy induction protocol in patients with unresectable LA-NSCLC.

## 2 Materials and methods

### 2.1 Patients

This was a retrospective cohort study of patients with histologically or cytologically documented stage III unresectable NSCLC (American Joint Committee on Cancer’s Cancer Staging Manual, 8th edition) who were treated at the Second Affiliated Hospital of Zhejiang University School of Medicine from May 1, 2018, to December 31, 2021. All patients underwent evaluation at our center by a multidisciplinary team comprising surgeons, oncologists, and radiologists, and were subsequently assessed as inoperable. Data were collected from patients with measurable lesions (according to Response Evaluation Criteria in Solid Tumors, RECIST 1.1 criteria) who received either chemoimmunotherapy induction therapy or chemo induction therapy before CRT. The exclusion criteria were: patients with EGFR mutation or ALK arrangement, surgical treatment, targeted therapy, induction ICI alone, concurrent ICI with RT or palliative treatment; lack of complete baseline information, radiologic imaging, or follow-up data; and disease progression after induction treatment. This study was approved by the Ethics Committee of the Second Affiliated Hospital, Zhejiang University School of Medicine (No. 2021-0420). The requirement for informed consent was waived because of the retrospective nature of the study.

### 2.2 Treatment strategy

**Figure 1:**
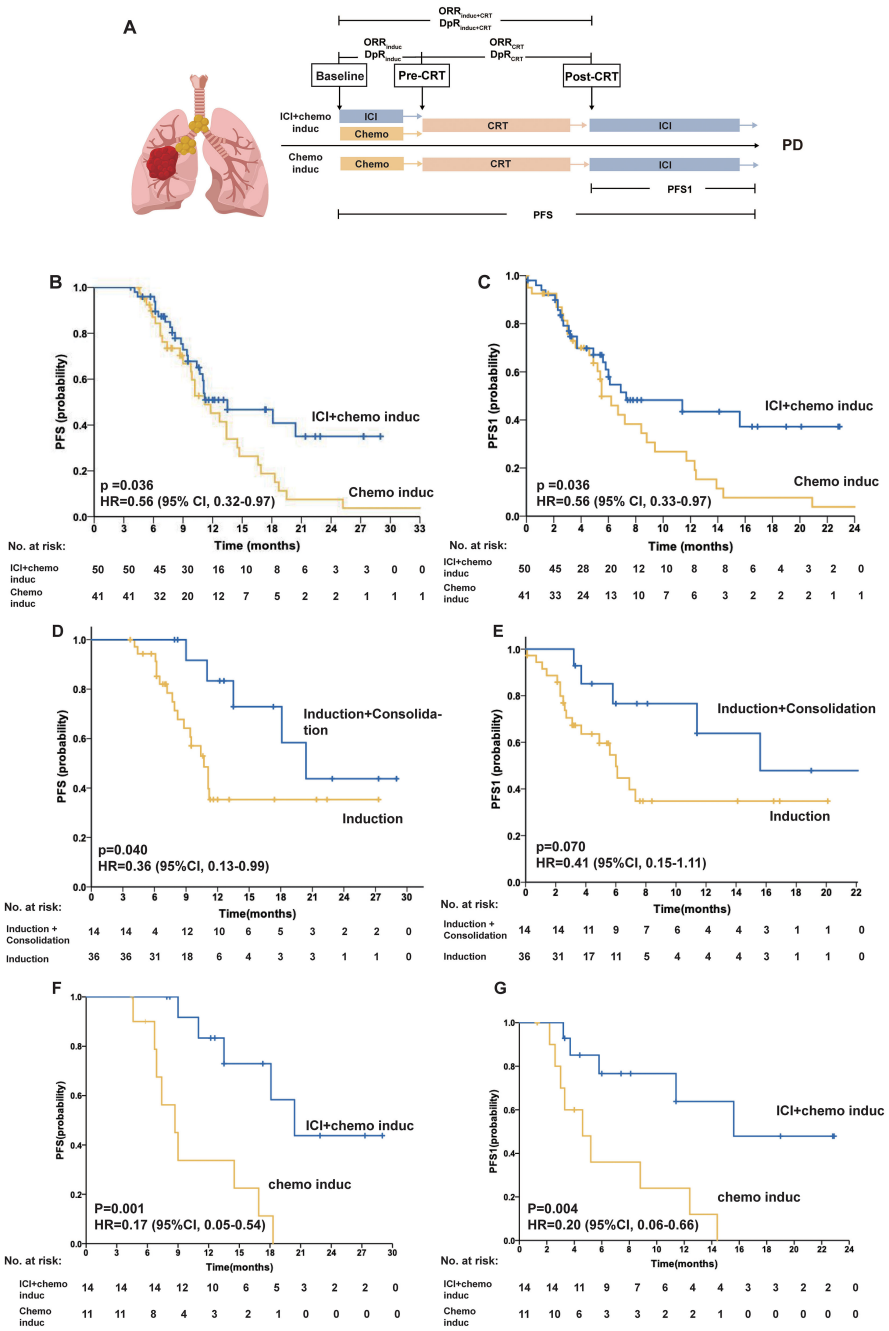
**(A)** The scheme of the study design. The tumor response was assessed prechemoradiotherapy (pre-CRT) and post-CRT. **(B–C)** PFS of the ICI + chemo induc and chemo induc groups. Kaplan–Meier curves of PFS **(B)** and PFS1 **(C)** for patients in the ICI + chemo induc (blue) and chemo induc (yellow) groups. **(D–E)** PFS of patients in the ICI + chemo induc group with or without consolidation treatment. Kaplan-Meier curves of PFS **(D)** and PFS1 **(E)** for patients who received induction+consolidation treatment (blue) and induction treatment only (yellow). **(F–G)** PFS of patients who underwent ICI consolidation treatment. Kaplan–Meier curves of PFS **(F)** and PFS1 **(G)** for patients in the ICI + chemo induc group (blue) and the chemo induc group (yellow). Ticks represent censored data. In the PFS analysis, data for patients without disease progression and still alive at the time of analysis were censored at their last assessment. chemo: Chemotherapy; CI: Confidence interval; CRT: Chemoradiation therapy; DpR: Depth of response; HR: Hazard ratio; ICI: Immune checkpoint inhibitor; Induc: Induction; ORR: Overall response rate; PD: Progressive disease; PFS: Progression-free survival; PFS1: Progression-free survival calculated from the end of CRT; post-CRT: Post-chemoradiotherapy; PR: Partial response; pre-CRT: Pre-chemoradiotherapy; SD: Stable disease.

Patients in the chemoimmunotherapy induction group (ICI+chemo induc group) received induction ICI plus chemotherapy before CRT with or without consolidation ICI. Patients in the chemotherapy induction group (chemo induc group) received chemotherapy induction before CRT with or without consolidation ICI (**Figure 1A**). The number of cycles of induction chemotherapy with or without ICI ranged from 2 to 3. Induction ICI consisted of pembrolizumab, camrelizumab, sintilimab, or tislelizumab. Induction chemotherapy consisted of carboplatin/cisplatin plus paclitaxel for squamous cell carcinoma and carboplatin/cisplatin plus pemetrexed for adenocarcinoma. CRT referred to intensity-modulated radiation therapy (IMRT) with a prescribed dose of 60–66 Gy administered concurrently with the same chemotherapy regimen used during the induction phase. The total RT dose was delivered using a 6-MV X-ray. The plan was normalized such that 100% of the prescription dose covered 95% of the target volume. RT was performed using either a three-dimensional treatment plan across multiple fixed fields or volumetric modulated arc therapy with a linear accelerator (Varian, Palo Alto, California, USA).

### 2.3 Clinical outcome measures and toxicity evaluation

Data on the baseline status, clinical manifestations, therapy, and imaging were obtained from the medical records. All patients underwent routine follow-up. The tumor response was assessed using chest computed tomography (CT), according to RECIST, version 1.1. AEs were assessed based on the Common Terminology Criteria for Adverse Events, version 5.0. The RT plan was evaluated using the Varian Eclipse 4.26 RT planning system.

The primary endpoint is progression-free survival (PFS), which was assessed from the initiation of induction treatment until the date of the first documented event of disease progression or until death without progression. PFS1 was assessed from the end of CRT until the date of the first documented event of disease progression or until death without progression. The overall response (ORR) and depth of response (DpR) were evaluated at the end of induction therapy (ORR_induc_, DpR_induc_) and CRT (ORR_induc+CRT_, DpR_induc+CRT_), respectively. In addition, ORR_CRT_ and DpR_CRT_ were determined as the ORR and DpR of the CRT, to evaluate the impact of induction therapy on CRT sensitivity. ORR was defined as a complete response (CR) plus a partial response (PR) according RECIST, version 1.1. DpR was defined as the percentage of maximal tumor reduction from baseline for the target lesions. Adverse events were graded based on Common Terminology Criteria for Adverse Events (CTCAE) version 5.

### 2.4 Statistical analysis

Treatment outcomes, including tumor responses, survival outcomes, and toxicity, were assessed in all patients. Data from the two groups were compared using a χ ^2^ or Fisher’s exact test for discrete variables and an unpaired t-test, Mann Whiney U test for continuous variables. Survival data were analyzed using the Kaplan–Meier method and compared using the log-rank test. Hazard ratios (HRs) were calculated using Cox’s proportional hazards models; results were reported with 95% confidence intervals (CIs). Analyses were performed using IBM SPSS version 26 software (IBM Corp., Armonk, NY, USA), The figure was generated by Figdraw (ResearchHome, Hangzhou, Zhejiang, China).

## 3 Results

### 3.1 Patient demographic and baseline characteristics

In this analysis, 91 patients were included, of whom 50 (55%) received chemoimmunotherapy induction therapy and 41 (45%) the chemo induction therapy. The majority of patients in the two groups were male (n = 83, 91.2%), and 28 (31.8%) were never-smokers. 26.8% of patients in ICI+chemo induc group and 28% in chemo induc group received ICI maintenance treatment. Baseline characteristics, including age, sex, stage, smoking status, histology type and median induction treatment duration was well balanced between the two groups (**Table 1**).

**Table 1:** Baseline characteristics.

	ICI+chemo induc group (n = 50)	chemo induc group (n = 41)	p value
**Age** **Median (P_25_, P_75_), years**	65 (51–78)	65 (50–79)	0.680
** <65, n (%)**	57 (51–63)	56 (50–64)	0.678
** ≥65, n (%)**	69 (61–78)	69 (65–79)	
**Sex, n (%)**			0.505
** Male**	47 (94.0)	36 (87.8)	
** Female**	3 (6.0)	5 (12.2)	
**Stage, n (%)**			0.541
** IIIA**	20 (40.0)	20 (48.8)	
** IIIB**	20 (40.0)	16 (39.0)	
** IIIC**	10 (20.0)	5 (12.2)	
**Smoking history, n (%)**			0.211
** Former**	20 (40.0)	18 (43.9)	
** Current**	11 (22.0)	14 (34.1)	
** Never**	19 (38.0)	9 (22.0)	
**Tumor histology, n (%)**			0.063
** Adenocarcinoma**	6 (26.1)	12 (29.3)	
** Squamous**	44 (64.7)	29 (70.7)	
**EGFR/ALK status, n (%)**			
** Positive**	0 (0.0%)	0 (0.0%)	–
** Negative**	50 (100%)	41 (100%)	
**Induction treatment duration, months, Median (P_25_, P_75_)**	2.9 (2.3, 4.0)	3.5 (2.6, 4.3)	0.237
**ICI maintenance treatment**			1.000
** Yes**	11 (26.8)	14 (28.0)	
** No**	30 (73.2)	36 (72.0)	

Data are presented as the median (P_25_, P_75_) or number (%); ICI, Immune checkpoint inhibitor; Induc, Induction; CRT, chemoradiation therapy.

### 3.2 Survival outcome

The median follow-up time was 17.8 months (95% CI, 14.7–20.9). The median PFS (mPFS) was significantly longer in the ICI+chemo induc group than in the chemo induc group (13.5 months vs. 11.2 months; HR, 0.56; 95% CI, 0.32–0.97; p = 0.036) (**Figure 1B**), as was the mPFS1 (7.3 months vs.5.5 months; HR, 0.56; 95% CI, 0.32–0.97; p = 0.036) (**Figure 1C**). In the ICI + chemo induc group, patients who underwent consolidation treatment had a significantly longer mPFS (20.4 months vs. 10.7 months; HR, 0.36; 95% CI, 0.13–0.99; p = 0.040) and a marginally longer mPFS1 (15.6 months vs. 6.0 months; HR, 0.41; 95% CI, 0.15–1.11; p = 0.071) (**Figures 1D, E**) than those who did not receive consolidation treatment. Among patients who received ICI consolidation treatment, inductive ICI + chemotherapy showed significantly better PFS than inductive chemotherapy (20.4 months vs. 9.7 months; HR, 0.17; 95% CI, 0.05–0.54; p= 0.001), and this finding was also observed for PFS1 (15.6 months vs. 4.6 months; HR, 0.20; 95% CI, 0.06–0.67; p = 0.004) (**Figures 1F, G**). Therefore, patients receiving inductive ICI + chemotherapy with consolidation ICI had the most favorable PFS/PFS1 outcomes.

### 3.3 Tumor response

**Figure 2:**
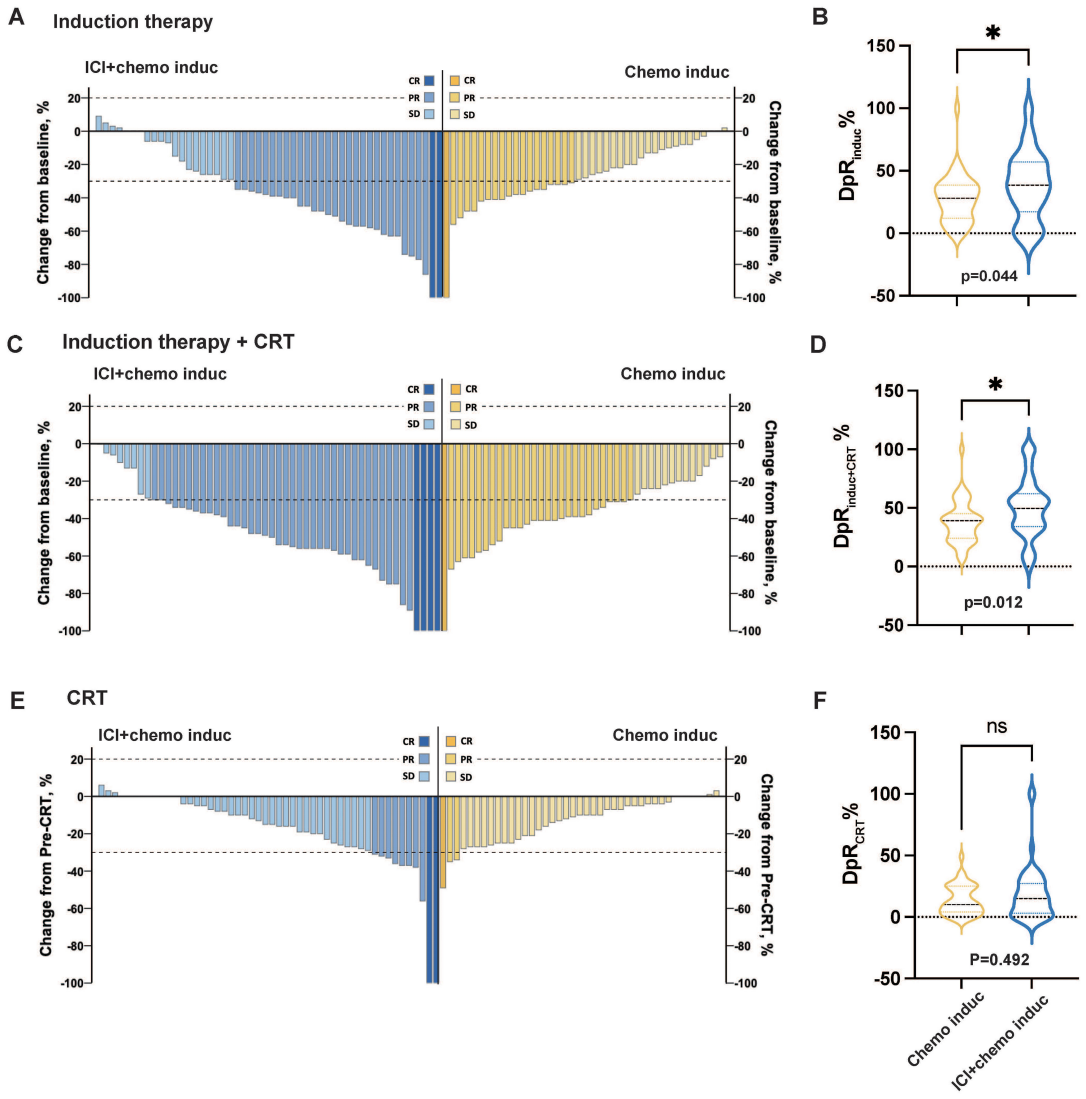
Change in the tumor burden in response to different treatment periods. **(A, C, E)** Change in the tumor burden in response to induction therapy **(A)**, induction therapy + CRT **(C)**, and CRT **(E)** for the ICI+chemo induc group (blue, n = 50) and chemo induc group (yellow, n = 41). **(B, D, F)** Depth of response (DpR) of induction therapy **(B)**, induction therapy + CRT **(D)**, and CRT **(F)** for the ICI+chemo induc group (blue, n = 50) and the chemo induc group (yellow, n=41). chemo, Chemotherapy; CR, Complete response; CRT, Chemoradiation therapy; DpR, Depth of response; ICI, Immune checkpoint inhibitor; Induc, Induction; ns, no significance; ORR, Overall response rate; PD, Progressive disease; PR, Partial response; pre-CRT, Pre-chemoradiotherapy; SD, Stable disease. ^*^p < 0.05.

The ORR and DpR at different treatment phases are summarized in **Table 2** and **Figure 2**. The ORR_induc+CRT_ was significantly higher in the ICI+chemo induc group than in the chemo induc group (84.0% vs. 65.9%, p = 0.044). Both ORR_induc_ and ORR_RT_ were numerically higher in the ICI+chemo induc group than the chemo induc group, but the difference was not statistically significant. The median DpR_induc_ (mDpR _induc_) was significantly higher in the ICI+chemo induc group than in the chemo induc group (38.5% vs. 28.0%, p = 0.044) (**Figures 2A, B**) as was the mDpR _induc+CRT_ (49.5% vs. 39.0%, p = 0.012) (**Figures 2C, D**), but the difference in the mDpR_CRT_ was not significant (ICI+chemo induc group vs. chemo induc group: 15.0% vs. 10.0%, p = 0.492) (**Figures 2E, F**).

**Table 2:** Tumor responses and efficacy assessments.

Treatment period	Outcomes	ICI + chemo induc group (n = 50)	Chemo induc group (n = 41)	p value
**Induction therapy**	Tumor response, *n* (%)			
	CR	2 (4.0)	1 (2.4)	
	PR	28 (56.0)	18 (43.9)	
	SD	20 (40.0)	22 (53.7)	
	ORR_induc_	60.0	46.3	0.193
	DpR_induc_ Median (P_25_, P_75_)	38.5 (17.3, 57.0)	28.0 (12.0, 38.5)	0.044
**Induction therapy + CRT**	Tumor response, *n* (%)			
	CR	4 (8.0)	1 (2.4)	
	PR	38 (76.0)	26 (63.4)	
	SD	8 (16.0)	14 (34.1)	
	ORR_induc+CRT_	84.0%	65.9%	0.044
	DpR_induc+CRT_ Median (P_25_, P_75_)	49.5 (34.0, 62.0)	39.0 (24.0,45.0)	0.012
**CRT**	Tumor response, *n* (%)			
	CR	2 (4.0)	0 (0)	
	PR	8 (16.0)	3 (7.3)	
	SD	40 (80.0)	38 (92.7)	
	ORR_CRT_	20.0	7.3	0.085
	DpR_CRT_ Median (P_25_, P_75_)	15.0 (3.0, 27.3)	10.0 (4.0, 25.0)	0.492

CR, Complete response; CRT, Chemoradiation therapy; DpR, Depth of response; ICI, Immune checkpoint inhibitor; ORR, Overall response rate; PD, Progressive disease; PR, Partial response; SD, Stable disease.

### 3.4 Safety profile

**Figure 3:**
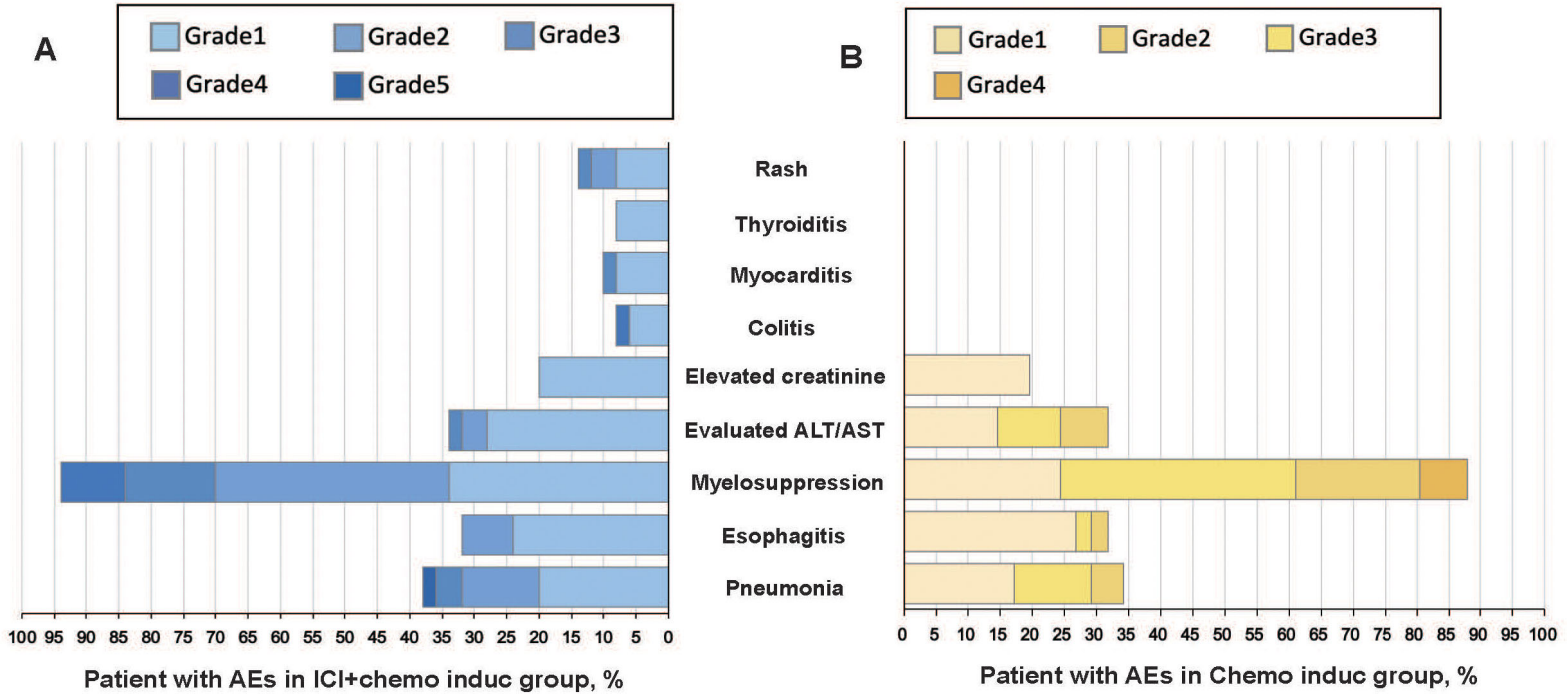
All-cause AEs grades 1–5 in the ICI + chemo induc **(A)** and chemo induc **(B)** groups. The color intensity reflects AE severity. AEs, Adverse events; ALT, Alanine aminotransferase; AST, Aspartate aminotransferases; Chemo, Chemotherapy; ICI, Immune checkpoint inhibitor.

Treatment-related AEs occurred in 48 patients (96.0%) in the ICI+chemo induc group and 40 patients (97.6%) in the chemo induc group. 17 patients (34.0%) in the ICI+chemo induc group and 16 (39.0%) in the chemo induc group had ≥ G3 AEs, of which myelosuppression was the most common in both groups. Pneumonitis (≥ G3) occurred in 3 patients (6.0%) in the ICI+chemo induc group and 2 (4.9%) in the chemo induc group. 1 patient in the ICI+chemo induc group died from pneumonitis, which was considered to be immune-related. The prevalence of esophagitis was comparable in the ICI+chemo induc and chemo induc groups (32% vs. 31.7%) and most cases were G1–2, except for one patient in the chemo induc group who suffered G3 esophagitis. The rates of all immune-related adverse events (irAEs) were < 10%, except for rash, which developed in 14% of patients in the ICI+chemo induc group but was generally mild (grade 1/2). 2 patients had ≥ G3 irAEs (1 colitis and 1 myocarditis), which in both cases resolved favorably (**Figure 3**).

### 3.5 Dosimetric parameters and preferred scheme

**Figure 4:**
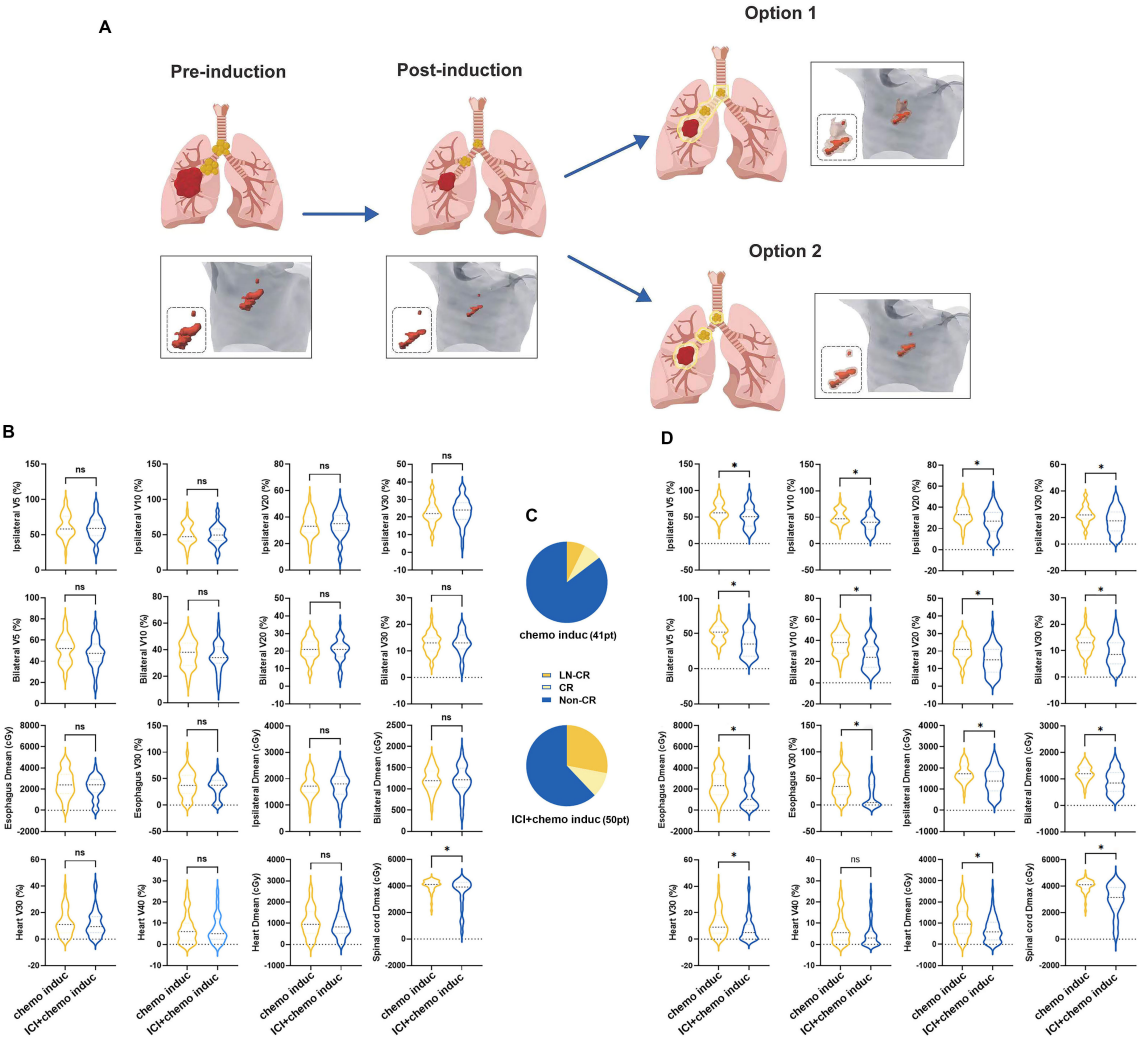
**(A)** RT was conducted according to the ESTRO ACROP guidelines after neoadjuvant therapy. Representative clinical target volume declined according to the two options in the ESTRO ACROP guidelines. **(B)** Exposure dose of normal tissue, including V5/V10/V20/V30 and Dmean of the ipsilateral lung and bilateral lungs, V30 and Dmean of the esophagus, V30/V40 and Dmean of the heart, and Dmax of the spinal cord. **(C)** Exposure dose of normal tissue calculated from the modified RT plan. **(D)** Efficacy evaluation before CRT. chemo, Chemotherapy; ns: no significance; CR, Complete response; Dmean, mean dose; ICI, Immune checkpoint inhibitor; Induc, Induction; LN, lymph node; pt, patient; V5/V10/V20/V30: percentage of lung volume exposed to >5/10/20/30 Gy. ^*^p < 0.05.

All patients successfully completed radiotherapy. The European Society for Radiotherapy and Oncology (ESTRO) Advisory Committee for Radiation Oncology Practice (ACROP) guidelines recommend two options regarding the clinical target volume (CTV) of the lymph nodes in NSCLC (12) (**Figure 4A**). In option 1 (lymph node stations), all of the pathologically affected lymph node stations are included while option 2 (geometric expansion) consists of geometric expansion of the nodal gross tumor volume (GTV) (13). In our center, option 1 is typically chosen. In this study, all patients were treated with option 1. The mean dose (Dmean) of the spinal cord was lower in patients in the ICI+chemo induc group than in the chemo induc group (**Figure 4B**) but there was no difference in the radiation dose distribution in normal tissue between the two groups, including V5/V10/V20/V30 (percentage of lung volume exposed to > 5/10/20/30 Gy) of the ipsilateral lung, bilateral V5/V10/V20/V30, the Dmean of the ipsilateral lung and bilateral lung, the Dmean and V30 of the esophagus, and the Dmean and V30/V40 of the heart (**Figure 4B**). This result is consistent with the comparable rates of toxicity and side effects in the two groups. However, the large reduction in tumor size achieved with the combination of ICIs+Chemo (**Figure 4C**) suggested that the target volume of RT could be reduced with option 2 (**Figure 4A**). We redefine the target volume and redesign the RT plan for the 91 patients according to option 2 of the ESTRO ACROP guidelines using the Varian Eclipse 4.26 RT planning system. We found that in the ICI+chemo induc group, the radiation dose to normal tissue, including lung, heart, esophagus, and spinal cord, would be dramatically reduced (**Figure 4D**) compared to the chemo induc group. This result suggested that if we delineate the target volume according to option 2, the toxicity of the ICI+chemo induc group might be lower.

## 4 Discussion

This is the first study to compare chemoimmunotherapy induction therapy with the chemo induction therapy before CRT in patients with LA-NSCLC. Our results showed that, compared to chemo induction therapy, chemoimmunotherapy remarkably improved the PFS, reduced the tumor burden, elevated the ORR, while did not increase the adverse events. Additionally, if we modified target volume of RT mapping strategy based on the remarkable reduction in tumor size triggered by induction therapy, the toxicity of the ICI+chemo induction group would be marked lower.

The treatment of locally advanced, unresectable NSCLC remains challenging (14). The current standard protocol consists of CRT followed by consolidation immunotherapy. However, in the real-world setting, LA-NSCLC patients usually bear high tumor burden and are not tolerated well to this standard strategy. Low treatment completion rate of the PACIFIC model because of the high toxicity caused by large RT target volume. The KEYNOTE-799 trial and other phase II trials, such as the ETOP NICOLAS trial of nivolumab plus CRT (15) and the DETERRED trial of atezolizumab plus CRT (16), explored the feasibility of concurrent ICI and CRT administration in patients with stage III LA-NSCLC. Despite the promising antitumor activity achieved with that approach, the incidence of treatment-related AEs increased. Recently, the PACIFIC-2 trial, a phase III study, evaluated the use of durvalumab initiated concurrently with concurrent chemoradiotherapy (cCRT), followed by consolidation durvalumab, compared to cCRT alone in patients with unresectable stage III NSCLC. The trial found that adding durvalumab did not improve outcomes compared to cCRT alone. The increased incidence of adverse events leading to death (13.7%) and the inclusion of 143 patients with unknown EGFR status may have influenced these results (11). Hence, it is urgent need to optimize the administration of ICI and CRT to maximize tumor control prior to CRT while minimizing AEs for patients LA-NSCLC, to improve the patients’ tolerability and consequently the survival benefit.

Our study demonstrated the feasibility of the integration of ICI with chemotherapy as induction therapy prior to CRT. In the ICI+chemo induc group, the ORR_induc_ during induction therapy was 60.0% and the mDpR_induc_ was 38.5%; both values were higher than those of the chemo induc group (ORR_induc_ 46.3%, mDpR_induc_ 28.0%). The ORR_induc_ was consistent with that reported in the Checkmate 816 trial (54% in the nivolizumab plus chemotherapy cohort vs. 37% in the chemotherapy alone cohort) for resectable NSCLC (9). In the retrospective study conducted by Wang et al., involving 75 patients with unresectable stage III NSCLC who received ICI and chemotherapy before CRT, the ORR achieved with induction therapy was 76.1% (17). These results indicate that the combination of ICIs and chemotherapy as induction therapy can effectively reduce the tumor burden and further reduce the RT target volume.

Theoretical research and clinical studies have shown that a larger irradiated area or a larger dose of RT tends to lead to worse outcomes, such as radiation pneumonitis (18–20) and radiation-induced heart disease (13, 20, 21). Both the PORT-C (22) and the Lung ART (23) studies reported increased cardiac toxicity and mortality with RT. A modified RT strategy able to reduce the toxicity and side effects of RT that does not compromise or even improves the curative effect is thus urgently needed. In our study, we conducted the RT plan according to the option 1 advise of the ESTRO ACROP guidelines and found that the Dmean of the spinal cord was lower in patients in the ICI+chemo induc group than in the chemo induc group. Due to the greater depth of tumor remission achieved with neoadjuvant therapy consisting of ICI and chemotherapy, we carried out another RT plan according to the option 2 advise of the ESTRO ACROP guidelines. The results concluded that a marked decrease in the accumulated dose at the lung, heart, esophagus, and spinal cord could be achieved in the ICI+chemo induc group. In the CRT phase, the mDpR_CRT_ in the ICI+chemo induc group was similar to that in the chemo induc group (15.0% vs. 10.0%), suggesting that adding ICI to the induction stage does not affect radiosensitivity. By the end of CRT, the ORR_induc+CRT_ (84.0% vs. 65.9%) and mDpR_induc+CRT_ (49.5% vs. 39.0%) were significantly higher in the ICI+chemo induc group than in the chemo induc group. The improved ORR is in line with that determined in the KEYNOTE 799 trial (24) and is comparable with the 86.7% ORR in the cohort of Wang et al (17).

A significant improvement in mPFS (13.5 vs 11.2 months) and mPFS1 (7.3 vs 5.5 months) was achieved in the ICI+chemo induc vs. the chemo induc group. The mPFS in the ICI+chemo induc group (13.5 months) was similar to that in the ETOP NICOLAS trial (12.7 months) (15), DETERRED trial (13.2 months) (16) and PACIFIC 2 trial (13.8m) (11). A previous study showed that a larger tumor volume at baseline is strongly linked to a worse survival outcome for patients with inoperable stage I-III NSCLC treated with RT (25). In the ICI+chemo induc group, the tumor burden after induction therapy was significantly reduced, which was likely to have contributed to the prolonged PFS. The increased mPFS1 may have been the result of the tailing effect of ICI, which suggests that an enhanced induction strategy, combining ICI with chemotherapy, can improve survival outcomes. Patients in the ICI+chemo induc group who received consolidation ICI therapy, had a longer mPFS and a longer mPFS1 than those who did not. Among the trials examining the modalities of ICI consolidation, the mPFS1 among patients with consolidation ICI therapy was better in our ICI+chemo induc group (15.6 months) than in patients in the GEMOSTONE-301 trial (9 months) and the PACIFIC-6 (10.9 months) trial and not worse than that of patients in the PACIFIC trial (16.8 months).These results indicated, even with a more enhanced induction strategy, consolidating ICI should still be essential for improving survival outcomes. The ongoing phase 3 KEYLYNK-012 trial (NCT04380636), investigating a regimen consisting of CRT combined with induction, concurrent, and consolidation pembrolizumab (26), may provide further insights into the role of different modalities combining ICI with CRT.

The safety issues of combined ICI and CRT are of great concern, as both ICI and RT are associated with pulmonary toxicities. A retrospective study showed that, compared to the administration of ICI after thoracic RT, administration during RT increased the incidence of pneumonia (27). In the PACIFIC trial, any grade pneumonia occurred in 33.9%, and ≥ G3 pneumonia in 3.4% of the patients who received ICI consolidation (28). The incidence of G3 pneumonia in real-world settings is much higher (14.3%) (6). In the KEYNOTE 799 trial, the occurrence rate of any grade and ≥ G3 pneumonia were 37.5% and 8.1% respectively (9, 24). In the retrospective study of Wang et al., the rate of any grade pneumonia was 48% and that of ≥ G3 pneumonia 9.3% (17). These results demonstrate the need for a modified protocol with lower toxicity. In our study, the incidence of ≥ G3 pneumonia in the ICI+chemo induc group was 6.0%, which was lower than that in the KEYNOTE 799 trial and slightly higher than that in the PACIFIC trial, indicating the importance of tumor downsizing before CRT and ICI cessation during CRT in controlling toxicity.

There were several limitations to this study. Firstly, the retrospective nature of the analysis should be considered when interpreting its conclusions, particularly due to the diverse treatment approaches employed, involving various ICIs. Previous research indicates that in the metastatic NSCLC setting, the use of four PD-1 inhibitors—camrelizumab, tislelizumab, sintilimab, and pembrolizumab—when combined with chemotherapy, significantly improves PFS compared to chemotherapy alone (29–32). Although we recognize the need for careful interpretation, the hazard ratios across these studies are similar. Therefore, we believe the impact of using different PD-1 inhibitors on our study’s outcomes might be minimal. The modified ICI and CRT combination protocol examined in this study provides the basis for larger, prospective studies. Second, the modified RT target volume plans were not actually implemented, although the indications were that, in the ICI+chemo induc group, the accumulated dose at vital organs could be markedly deceased. The ability of this approach to reduce RT toxicity as expected and its potential impact on survival should be verified in a real-world cohort. Finally, overall survival could not be determined, and the median overall survival was not yet reached during the study period, due to the relatively short follow-up time.

## 5 Conclusions

In conclusion, the incorporating ICI to induction chemotherapy before concurrent CRT and followed by consolidation ICI was shown to remarkably reduce the tumor burden, improve the PFS, and potentially lower toxicity if an improved target volume mapping strategy were implemented. This modified approach may improve the survival outcome of patients with unresectable stage III NSCLC. Further large multicenter random controlled trials are therefore warranted.

## Data Availability

The raw data supporting the conclusions of this article will be made available by the authors, without undue reservation.
